# 1-(2-Hy­droxy­eth­yl)-3-(3-meth­oxy­phen­yl)thio­urea

**DOI:** 10.1107/S1600536810034665

**Published:** 2010-09-04

**Authors:** Hyeong Choi, Yong Suk Shim, Byung Hee Han, Sung Kwon Kang, Chang Keun Sung

**Affiliations:** a Department of Chemistry, Chungnam National University, Daejeon 305-764, Republic of Korea; b Department of Food Science and Technology, Chungnam National University, Daejeon 305-764, Republic of Korea

## Abstract

In the title compound, C_10_H_14_N_2_O_3_S, the 3-meth­oxy­phenyl unit is almost planar, with an r.m.s. deviation of 0.013 Å. The dihedral angle between the benzene ring and the plane of the thio­urea unit is 62.57 (4)°. In the crystal, N—H⋯O and O—H⋯S hydrogen bonds link the mol­ecules into a three-dimensional network.

## Related literature

For general background to melanin, see: Ha *et al.* (2007 ▶). For the development of potent inhibitory agents of tyrosinase, see: Kojima *et al.* (1995 ▶); Cabanes *et al.* (1994 ▶); Casanola-Martin *et al.* (2006 ▶); Son *et al.* (2000 ▶); Iida *et al.* (1995 ▶). For thio­urea derivatives, see: Thanigaimalai *et al.* (2010 ▶); Klabunde *et al.* (1998 ▶); Criton (2006 ▶); Daniel (2006 ▶); Yi *et al.* (2009 ▶); Liu *et al.* (2009 ▶).
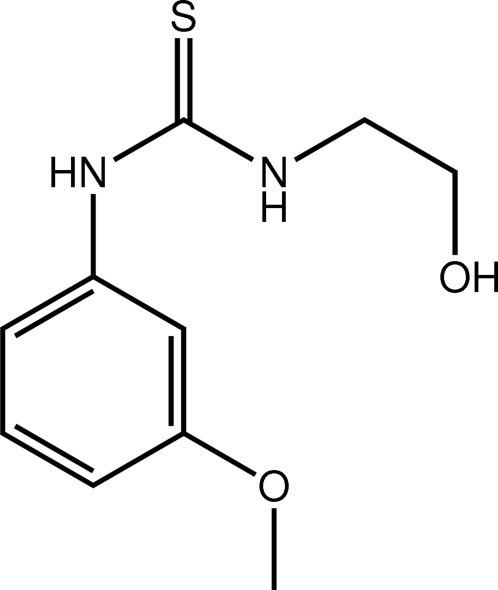


## Experimental

### 

#### Crystal data

C_10_H_14_N_2_O_2_S
*M* *_r_* = 226.29
Monoclinic, 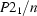

*a* = 10.9894 (3) Å
*b* = 8.0759 (2) Å
*c* = 12.8067 (4) Å
β = 102.920 (1)°
*V* = 1107.81 (5) Å^3^
*Z* = 4
Mo *K*α radiation
μ = 0.27 mm^−1^
*T* = 296 K
0.37 × 0.21 × 0.2 mm

#### Data collection

Bruker SMART CCD area-detector diffractometer
8965 measured reflections
2478 independent reflections
2013 reflections with *I* > 2σ(*I*)
*R*_int_ = 0.059

#### Refinement

*R*[*F* ^2^ > 2σ(*F* ^2^)] = 0.038
*wR*(*F* ^2^) = 0.107
*S* = 1.08
2478 reflections
148 parameters
H atoms treated by a mixture of independent and constrained refinement
Δρ_max_ = 0.22 e Å^−3^
Δρ_min_ = −0.36 e Å^−3^

### 

Data collection: *SMART* (Bruker, 2002 ▶); cell refinement: *SAINT* (Bruker, 2002 ▶); data reduction: *SAINT*; program(s) used to solve structure: *SHELXS97* (Sheldrick, 2008 ▶); program(s) used to refine structure: *SHELXL97* (Sheldrick, 2008 ▶); molecular graphics: *DIAMOND* (Brandenburg, 2010 ▶); software used to prepare material for publication: *WinGX* (Farrugia, 1999 ▶).

## Supplementary Material

Crystal structure: contains datablocks global, I. DOI: 10.1107/S1600536810034665/tk2706sup1.cif

Structure factors: contains datablocks I. DOI: 10.1107/S1600536810034665/tk2706Isup2.hkl

Additional supplementary materials:  crystallographic information; 3D view; checkCIF report

## Figures and Tables

**Table 1:** Hydrogen-bond geometry (Å, °)

*D*—H⋯*A*	*D*—H	H⋯*A*	*D*⋯*A*	*D*—H⋯*A*
N7—H7⋯O13^i^	0.824 (19)	2.059 (19)	2.8619 (16)	164.6 (17)
N10—H10⋯O14^ii^	0.817 (19)	2.316 (19)	3.0877 (17)	157.8 (15)
O13—H13⋯S9^iii^	0.81 (2)	2.47 (2)	3.2532 (14)	163 (2)

Symmetry codes: (i) 
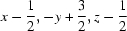
; (ii) 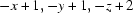; (iii) 
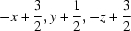
.

**Fig. 1.**
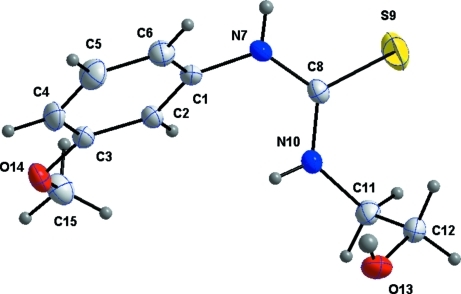
Molecular structure of (I), showing the atom-numbering scheme and 50% probability ellipsoids.

**Fig. 2.**
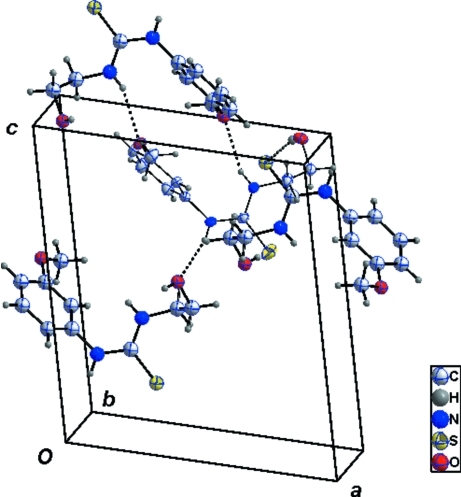
Part of the crystal structure of (I), connections between molecules by intermolecular N—H···O and O—H···S hydrogen bonds (dashed lines).

## supplementary crystallographic information

### Comment

Melanin is the pigment responsible for the color of human skin and it is formed through a series of oxidative reactions in the presence of key enzyme tyrosinase (Ha *et al.*, 2007) that converts tyrosine into melanin. It is secreted by melanocyte cells distributed in the basal layer of the dermis. Its role is to protect the skin from ultraviolet (UV) damage by absorbing the UV sunlight and removing reactive oxygen species. Therefore, its inhibitors are target molecules for developing anti-pigmentation agents. Numerous potential tyrosinase inhibitors have been discovered from natural and synthetic sources, such as ascorbic acid (Kojima *et al.*, 1995), kojic acid (Cabanes *et al.*, 1994), arbutin (Casanola-Martin *et al.*, 2006) and tropolone (Son *et al.*, 2000; Iida *et al.*, 1995). Some thiourea derivatives, such as phenylthiourea (Thanigaimalai *et al.*, 2010; Klabunde *et al.*, 1998; Criton, 2006), alkylthiourea (Daniel, 2006), thiosemicarbazone (Yi *et al.*, 2009) and thiosemicarbazide (Liu *et al.*, 2009) have been also described. However, only few of the reported compounds are used in medicinal and cosmetic products because of their lower activities, poor skin penetration, or serious side effects. Consequently, there is still a need to search and develop novel tyrosinase inhibitors with better activities together with lower side effects. To complement the inadequacy of current whitening agents and maximize the effect of inhibition of melanin creation, we have synthesized the title compound, (I), from the reaction of 3-methoxyphenyl isothiocyanate and ethanolamine under ambient condition. Here, the crystal structure of (I) is described (Fig. 1).

The 3-methoxyphenyl unit is essentially planar, with a r.m.s. deviation of 0.013 Å from the corresponding least-squares plane defined by the eight constituent atoms. The dihedral angle between the benzene ring and the plane of the thiourea moiety is 62.57 (4) °. In the crystal, N—H···O and O—H···S hydrogen bonds link the molecules into a 3-D network (Fig. 2, Table 1). The H atoms of the NH groups of thiourea are positioned *anti* to each other.

### Experimental

Ethanolamine and 3-methoxyphenyl isothiocyanate were purchased from Sigma Chemical Co. Solvents used for organic synthesis were distilled before use. All other chemicals and solvents were of analytical grade and were used without further purification. The title compound (I) was prepared from the reaction of 3-methoxyphenyl isothiocyanate (0.4 ml, 1 mmol) with ethanolamine (0.2 ml, 1.2 mmol) in acetonitrile (6 ml). The reaction was completed within 30 min at room temperature. The reaction mixture was filtered and washed with dry *n*-hexane. Removal of the solvent under vacuum gave a white solid (80%, m.p. 398 K). Single crystals were obtained by slow evaporation of the ethanol solution held at room temperature.

### Refinement

The H atoms of the NH and OH groups were located in a difference Fourier map and refined freely. The remaining H atoms were positioned geometrically and refined using a riding model with C—H = 0.93–0.97 Å, and with *U*_iso_(H) = 1.2*U*_eq_ (C) for aromatic- and methylene-H, and 1.5*U*_eq_(C) for methyl-H atoms.

### Crystal data

**Table:**

C_10_H_14_N_2_O_2_S	*F*(000) = 480
*M**_r_* = 226.29	*D*_x_ = 1.357 Mg m^−^^3^
Monoclinic, *P*2_1_/*n*	Mo *K*α radiation, λ = 0.71073 Å
Hall symbol: -P 2yn	Cell parameters from 4441 reflections
*a* = 10.9894 (3) Å	θ = 2.8–28.1°
*b* = 8.0759 (2) Å	µ = 0.27 mm^−^^1^
*c* = 12.8067 (4) Å	*T* = 296 K
β = 102.920 (1)°	Block, colorless
*V* = 1107.81 (5) Å^3^	0.37 × 0.21 × 0.2 mm
*Z* = 4	

### Data collection

**Table:**

Bruker SMART CCD area-detector diffractometer	*R*_int_ = 0.059
φ and ω scans	θ_max_ = 27.5°, θ_min_ = 2.2°
8965 measured reflections	*h* = −10→14
2478 independent reflections	*k* = −4→10
2013 reflections with *I* > 2σ(*I*)	*l* = −15→15

### Refinement

**Table:**

Refinement on *F*^2^	0 restraints
Least-squares matrix: full	H atoms treated by a mixture of independent and constrained refinement
*R*[*F*^2^ > 2σ(*F*^2^)] = 0.038	*w* = 1/[σ^2^(*F*_o_^2^) + (0.0621*P*)^2^ + 0.0837*P*] where *P* = (*F*_o_^2^ + 2*F*_c_^2^)/3
*wR*(*F*^2^) = 0.107	(Δ/σ)_max_ = 0.001
*S* = 1.08	Δρ_max_ = 0.22 e Å^−^^3^
2478 reflections	Δρ_min_ = −0.36 e Å^−^^3^
148 parameters	

### Special details

**Table:**

Geometry. All e.s.d.'s (except the e.s.d. in the dihedral angle between two l.s. planes) are estimated using the full covariance matrix. The cell e.s.d.'s are taken into account individually in the estimation of e.s.d.'s in distances, angles and torsion angles; correlations between e.s.d.'s in cell parameters are only used when they are defined by crystal symmetry. An approximate (isotropic) treatment of cell e.s.d.'s is used for estimating e.s.d.'s involving l.s. planes.

### Fractional atomic coordinates and isotropic or equivalent isotropic displacement parameters (Å^2^)

**Table:**

	*x*	*y*	*z*	*U*_iso_*/*U*_eq_	
C1	0.47197 (11)	0.63459 (15)	0.77190 (10)	0.0330 (3)	
C2	0.46149 (11)	0.49103 (15)	0.83014 (10)	0.0330 (3)	
H2	0.5124	0.4001	0.8268	0.04*	
C3	0.37377 (11)	0.48652 (16)	0.89296 (10)	0.0350 (3)	
C4	0.29844 (13)	0.62354 (19)	0.89796 (13)	0.0463 (4)	
H4	0.2409	0.621	0.9413	0.056*	
C5	0.30915 (14)	0.7629 (2)	0.83859 (14)	0.0532 (4)	
H5	0.2576	0.8534	0.8412	0.064*	
C6	0.39582 (13)	0.76975 (19)	0.77507 (13)	0.0464 (4)	
H6	0.4027	0.864	0.7351	0.056*	
N7	0.55784 (11)	0.64047 (14)	0.70312 (10)	0.0377 (3)	
H7	0.5267 (16)	0.658 (2)	0.6393 (16)	0.051 (5)*	
C8	0.68277 (12)	0.62256 (14)	0.73143 (11)	0.0341 (3)	
S9	0.76633 (4)	0.61967 (5)	0.63478 (3)	0.05057 (15)	
N10	0.73496 (11)	0.60801 (14)	0.83569 (10)	0.0364 (3)	
H10	0.6920 (16)	0.6201 (17)	0.8796 (14)	0.040 (4)*	
C11	0.86769 (12)	0.57980 (18)	0.87843 (13)	0.0426 (3)	
H11A	0.9014	0.5185	0.8262	0.051*	
H11B	0.8782	0.5124	0.9425	0.051*	
C12	0.94037 (12)	0.73800 (18)	0.90533 (12)	0.0443 (3)	
H12A	1.0289	0.7127	0.9243	0.053*	
H12B	0.9253	0.8091	0.8427	0.053*	
O13	0.90658 (11)	0.82309 (17)	0.99133 (9)	0.0553 (3)	
H13	0.865 (2)	0.906 (3)	0.973 (2)	0.092 (8)*	
O14	0.35374 (10)	0.35339 (12)	0.95322 (9)	0.0469 (3)	
C15	0.40934 (16)	0.1998 (2)	0.93648 (14)	0.0546 (4)	
H15A	0.3879	0.1178	0.9836	0.082*	
H15B	0.4984	0.2123	0.9511	0.082*	
H15C	0.3793	0.1655	0.8635	0.082*	

### Atomic displacement parameters (Å^2^)

**Table:**

	*U*^11^	*U*^22^	*U*^33^	*U*^12^	*U*^13^	*U*^23^
C1	0.0284 (6)	0.0451 (7)	0.0251 (7)	−0.0024 (5)	0.0048 (5)	0.0000 (5)
C2	0.0314 (6)	0.0387 (6)	0.0299 (7)	−0.0006 (5)	0.0092 (5)	−0.0023 (5)
C3	0.0319 (6)	0.0455 (7)	0.0283 (7)	−0.0063 (5)	0.0079 (5)	−0.0037 (5)
C4	0.0348 (7)	0.0624 (9)	0.0460 (9)	0.0024 (6)	0.0182 (6)	−0.0052 (6)
C5	0.0439 (8)	0.0553 (9)	0.0624 (11)	0.0153 (7)	0.0162 (7)	0.0019 (7)
C6	0.0431 (7)	0.0482 (7)	0.0479 (9)	0.0066 (6)	0.0099 (6)	0.0096 (6)
N7	0.0354 (6)	0.0528 (7)	0.0256 (7)	−0.0022 (5)	0.0084 (5)	0.0063 (5)
C8	0.0372 (7)	0.0328 (6)	0.0348 (8)	−0.0040 (5)	0.0134 (6)	0.0013 (5)
S9	0.0494 (2)	0.0667 (3)	0.0431 (3)	−0.01090 (17)	0.02646 (18)	−0.00218 (16)
N10	0.0307 (5)	0.0481 (6)	0.0321 (7)	−0.0006 (4)	0.0105 (5)	0.0030 (5)
C11	0.0339 (7)	0.0469 (7)	0.0472 (9)	0.0064 (5)	0.0092 (6)	0.0105 (6)
C12	0.0313 (6)	0.0598 (8)	0.0420 (8)	−0.0017 (6)	0.0089 (6)	0.0081 (6)
O13	0.0546 (7)	0.0724 (8)	0.0347 (6)	−0.0015 (6)	0.0011 (5)	−0.0046 (5)
O14	0.0517 (6)	0.0520 (6)	0.0440 (6)	−0.0067 (4)	0.0255 (5)	0.0017 (4)
C15	0.0634 (10)	0.0484 (8)	0.0561 (11)	−0.0017 (7)	0.0223 (8)	0.0085 (7)

### Geometric parameters (Å, °)

**Table:**

C1—C6	1.3815 (18)	C8—S9	1.6983 (14)
C1—C2	1.3972 (17)	N10—C11	1.4567 (17)
C1—N7	1.4284 (18)	N10—H10	0.817 (19)
C2—C3	1.3874 (18)	C11—C12	1.505 (2)
C2—H2	0.93	C11—H11A	0.97
C3—O14	1.3696 (16)	C11—H11B	0.97
C3—C4	1.392 (2)	C12—O13	1.4163 (19)
C4—C5	1.378 (2)	C12—H12A	0.97
C4—H4	0.93	C12—H12B	0.97
C5—C6	1.385 (2)	O13—H13	0.81 (2)
C5—H5	0.93	O14—C15	1.4200 (19)
C6—H6	0.93	C15—H15A	0.96
N7—C8	1.3471 (17)	C15—H15B	0.96
N7—H7	0.824 (19)	C15—H15C	0.96
C8—N10	1.3353 (18)		
			
C6—C1—C2	121.14 (12)	C8—N10—C11	124.03 (13)
C6—C1—N7	118.68 (12)	C8—N10—H10	119.6 (12)
C2—C1—N7	120.10 (11)	C11—N10—H10	116.3 (12)
C3—C2—C1	118.83 (11)	N10—C11—C12	112.87 (11)
C3—C2—H2	120.6	N10—C11—H11A	109
C1—C2—H2	120.6	C12—C11—H11A	109
O14—C3—C2	124.57 (12)	N10—C11—H11B	109
O14—C3—C4	115.21 (12)	C12—C11—H11B	109
C2—C3—C4	120.22 (13)	H11A—C11—H11B	107.8
C5—C4—C3	119.92 (14)	O13—C12—C11	111.83 (12)
C5—C4—H4	120	O13—C12—H12A	109.2
C3—C4—H4	120	C11—C12—H12A	109.2
C4—C5—C6	120.77 (14)	O13—C12—H12B	109.2
C4—C5—H5	119.6	C11—C12—H12B	109.2
C6—C5—H5	119.6	H12A—C12—H12B	107.9
C1—C6—C5	119.10 (14)	C12—O13—H13	113.9 (18)
C1—C6—H6	120.5	C3—O14—C15	118.15 (11)
C5—C6—H6	120.5	O14—C15—H15A	109.5
C8—N7—C1	127.20 (12)	O14—C15—H15B	109.5
C8—N7—H7	117.2 (13)	H15A—C15—H15B	109.5
C1—N7—H7	115.6 (13)	O14—C15—H15C	109.5
N10—C8—N7	117.55 (13)	H15A—C15—H15C	109.5
N10—C8—S9	123.11 (10)	H15B—C15—H15C	109.5
N7—C8—S9	119.34 (11)		

### Hydrogen-bond geometry (Å, °)

**Table:**

*D*—H···*A*	*D*—H	H···*A*	*D*···*A*	*D*—H···*A*
N7—H7···O13^i^	0.824 (19)	2.059 (19)	2.8619 (16)	164.6 (17)
N10—H10···O14^ii^	0.817 (19)	2.316 (19)	3.0877 (17)	157.8 (15)
O13—H13···S9^iii^	0.81 (2)	2.47 (2)	3.2532 (14)	163 (2)

Symmetry codes: (i) *x*−1/2, −*y*+3/2, *z*−1/2; (ii) −*x*+1, −*y*+1, −*z*+2; (iii) −*x*+3/2, *y*+1/2, −*z*+3/2.
